# Supplementary material for: Effect of mediolateral leg perturbations on walking balance in people with chronic stroke: A randomized controlled trial
Source: PLoS One. 2024 Oct 8;19(10):e0311727. doi: 10.1371/journal.pone.0311727 (PMC11460716; doi:10.1371/journal.pone.0311727)
Supplement: S1 Appendix — (DOCX) [file pone.0311727.s002.docx]

**Effect of mediolateral leg perturbations on walking balance in people with chronic stroke: a randomized controlled trial**

**Appendix A**

The purpose of this study was to investigate whether the modulation of mediolateral paretic step location can be strengthened through repeated exposure to a novel mechanical environment. Our focus on steps taken with the paretic leg was justified by prior findings that step width modulation is significantly reduced for paretic steps [1], and that the mediolateral foot placement modulation of paretic steps is linked to clinical measures of balance [2]. However, the design of our force-field applies forces to both the paretic and non-paretic legs during walking. Therefore, we here assessed whether the effects of the three force-field control modes (Control, Assistive, Perturbing) on paretic step modulation were also observed for non-paretic steps. As in the main text, the results are presented in Figures S1-2, with corresponding statistical values in Table S1.

In brief, the effects on non-paretic steps were similar to the effects on paretic steps, although to a somewhat lesser extent. Specifically, neither non-paretic step width modulation (ρ_SW_; Fig. S1A, E) nor non-paretic foot placement modulation (ρ_FP_; Fig. S1B, F) changed significantly from their Pre-Intervention value at any subsequent time point in either the Control group or the Assistive group. In contrast, both metrics increased significantly at several later time points for the Perturbing group. Compared to the Control group, the Perturbing group exhibited significantly larger increases in non-paretic ρ_FP_ at Week 8. Final pelvis displacement modulation for non-paretic steps (ρ_PD_; Fig. S1C, G) did not change significantly across assessments for any intervention group. The average mediolateral foot placement location for steps taken with the non-paretic leg decreased significantly over time only for the Perturbing group, although this decrease did not remain significant for the Follow-up assessment (Fig. S1D, H). Despite this decrease in foot placement location over time, the Perturbing group still placed their non-paretic foot farther laterally than the Control group for most assessment time points.

The biomechanical analyses presented in the main text focused on the relationship between pelvis dynamics at the start of a step and body configuration (e.g., step width, foot placement) at the end of a step. Such metrics quantify the extent to which participants adjust their body configuration over the course of a step. Alternatively, one could characterize the relationship between pelvis dynamics at the end of the step and body configuration at the same point in time [3], metrics that quantify the extent to which any within-step adjustments appropriately re-configured the body. Here we present such metrics for both paretic and non-paretic steps.

Even when solely focusing on this later point in the gait cycle, the vast majority of the observed biomechanical changes occurred in the Perturbing group. At the end of paretic steps, the Perturbing group exhibited significant increases in paretic ρ_SW_ (Fig. S2A, E) and paretic ρ_FP_ (Fig. S2B, F) relative to baseline for most time points. No changes in these metrics were present for the Control group, and only one instance of a significant change in paretic ρ_SW_ was observed in the Assistive group. At the end of non-paretic steps, the Perturbing group exhibited significant increases in non-paretic ρ_SW_ (Fig. S2C, G) and non-paretic ρ_FP_ (Fig. S2D,H) at both the Week 8 and Post-Intervention time points. Neither the Control nor Assistive groups exhibited any significant changes in these metrics.

Overall, these supplementary results are consistent with the findings in the main text. Repeated exposure to targeted perturbations caused numerous examples of increased modulation of step width and mediolateral foot placement, while a control condition and foot placement assistance had only minimal effects on these biomechanical measures of gait behavior.

**Figure S1.** Effects of the three force-field intervention groups on modulation of mediolateral non-paretic step location. The figure follows the same structure as the corresponding figures in the main text.

**Figure S2.** Effects of the three force-field intervention groups on step location, based on pelvis dynamics at the end of the step. The figure follows the same structure as the corresponding figures in the main text.

**Table S1. Tertiary outcome measures**

| **Outcome Metrics** | Comparisons to baseline within each intervention group | | | Comparisons of metric values with Control group | | Comparison of metric changes with Control group | |
| --- | --- | --- | --- | --- | --- | --- | --- |
|  | **Control** | **Assistive** | **Perturbing** | **Assistive** | **Perturbing** | **Assistive** | **Perturbing** |
|  | t (p) | t (p) | t (p) | t (p) | t (p) | t (p) | t (p) |
| **Nonparetic ρ_SW_** | | | | | | | |
| Baseline |  |  |  | -0.95 (0.34) | -1.56 (0.12) |  |  |
| Week 4 | 0.08 (0.94) | 0.15 (0.88) | 0.22 (0.83) | -0.91 (0.37) | -1.48 (0.14) | 0.04 (0.96) | 0.08 (0.93) |
| Week 8 | -0.37 (0.72) | 0.74 (0.46) | **2.53 (0.013)** | -0.17 (0.86) | 0.39 (0.70) | 0.78 (0.44) | 1.94 (0.05) |
| Week 12 | -0.38 (0.71) | 0.49 (0.62) | **2.54 (0.012)** | -0.35 (0.72) | 0.36 (0.72) | 0.61 (0.54) | 1.96 (0.05) |
| Follow-up | -0.61 (0.54) | -0.70 (0.48) | 1.88 (0.06) | -1.00 (0.32) | -0.07 (0.94) | -0.05 (0.96) | 1.69 (0.09) |
| **Nonparetic ρ_FP_** | | | | | | | |
| Baseline |  |  |  | -0.74 (0.46) | -1.65 (0.10) |  |  |
| Week 4 | 0.84 (0.40) | 1.23 (0.22) | 1.63 (0.11) | -0.52 (0.60) | -1.25 (0.21) | 0.24 (0.81) | 0.44 (0.66) |
| Week 8 | 0.16 (0.88) | 1.48 (0.14) | **4.13 (<0.0001)** | 0.08 (0.94) | 0.69 (0.49) | 0.91 (0.36) | **2.60 (0.010)** |
| Week 12 | 0.75 (0.45) | 0.89 (0.37) | **3.41 (0.0008)** | -0.67 (0.50) | -0.15 (0.88) | 0.08 (0.94) | 1.68 (0.09) |
| Follow-up | -0.59 (0.56) | 0.38 (0.70) | **2.20 (0.030)** | -0.18 (0.86) | -0.10 (0.92) | 0.69 (0.49) | 1.89 (0.06) |
| **Nonparetic ρ_PD_** | | | | | | | |
| Baseline |  |  |  | -1.36 (0.18) | -0.12 (0.91) |  |  |
| Week 4 | -0.90 (0.37) | 0.56 (0.58) | -1.30 (0.20) | -0.19 (0.85) | -0.31 (0.76) | 1.04 (0.30) | -0.17 (0.86) |
| Week 8 | -1.01 (0.31) | 0.43 (0.67) | -0.39 (0.70) | -0.20 (0.84) | 0.45 (0.65) | 1.03 (0.31) | 0.51 (0.61) |
| Week 12 | -1.20 (0.23) | -0.10 (0.92) | -1.02 (0.31) | -0.47 (0.64) | 0.14 (0.89) | 0.79 (0.43) | 0.23 (0.82) |
| Follow-up | -0.42 (0.67) | -0.70 (0.49) | -1.19 (0.24) | -1.55 (0.12) | -0.61 (0.55) | -0.18 (0.86) | -0.46 (0.64) |
| **Nonparetic mediolateral foot placement** | | | | | | | |
| Baseline |  |  |  | 0.52 (0.61) | **2.49 (0.016)** |  |  |
| Week 4 | -1.49 (0.14) | 0.66 (0.51) | **-2.70 (0.0076)** | 1.48 (0.14) | **2.07 (0.043)** | 1.53 (0.13) | -0.66 (0.51) |
| Week 8 | -1.36 (0.17) | -0.82 (0.41) | **-3.11 (0.0023)** | 0.78 (0.44) | 1.85 (0.07) | 0.41 (0.68) | -1.02 (0.31) |
| Week 12 | -1.36 (0.18) | -1.05 (0.30) | **-2.44 (0.016)** | 0.67 (0.50) | **2.12 (0.038)** | 0.25 (0.80) | -0.58 (0.56) |
| Follow-up | -0.63 (0.53) | -0.80 (0.43) | -1.64 (0.10) | 0.45 (0.65) | **2.1 (0.041)** | -0.10 (0.92) | -0.60 (0.55) |
| **Paretic step end ρ_SW_** | | | | | | | |
| Baseline |  |  |  | -0.48 (0.63) | -1.60 (0.11) |  |  |
| Week 4 | -0.41 (0.69) | 1.64 (0.10) | **2.90 (0.0043)** | 0.68 (0.50) | 0.18 (0.86) | 1.43 (0.15) | **2.21 (0.028)** |
| Week 8 | -0.34 (0.73) | **2.19 (0.030)** | **3.35 (0.001)** | 0.95 (0.35) | 0.38 (0.70) | 1.77 (0.08) | **2.46 (0.015)** |
| Week 12 | -0.39 (0.70) | -0.04 (0.97) | **2.75 (0.0066)** | -0.27 (0.79) | 0.10 (0.92) | 0.25 (0.80) | **2.11 (0.037)** |
| Follow-up | -0.86 (0.39) | 0.34 (0.73) | 1.69 (0.09) | 0.24 (0.81) | -0.12 (0.90) | 0.86 (0.39) | 1.76 (0.08) |
| **Paretic step end ρ_FP_** | | | | | | | |
| Baseline |  |  |  | 0.37 (0.71) | -0.24 (0.81) |  |  |
| Week 4 | 0.95 (0.34) | 0.27 (0.79) | **2.20 (0.029)** | -0.06 (0.95) | 0.40 (0.69) | -0.50 (0.62) | 0.73 (0.47) |
| Week 8 | -0.09 (0.93) | 1.40 (0.16) | **3.89 (0.0001)** | 1.28 (0.21) | **2.06 (0.043)** | 1.03 (0.30) | **2.63 (0.0095)** |
| Week 12 | 0.26 (0.80) | -0.58 (0.56) | **3.38 (0.0009)** | -0.14 (0.89) | 1.55 (0.13) | -0.59 (0.56) | **2.03 (0.044)** |
| Follow-up | 0.09 (0.93) | -0.34 (0.73) | **2.49 (0.014)** | 0.10 (0.92) | 1.18 (0.24) | -0.30 (0.76) | 1.57 (0.12) |
| **Nonparetic step end ρ_SW_** | | | | | | | |
| Baseline |  |  |  | -0.23 (0.82) | -1.20 (0.23) |  |  |
| Week 4 | 0.80 (0.43) | 0.59 (0.56) | 0.38 (0.71) | -0.40 (0.67) | -1.58 (0.12) | -0.16 (0.87) | -0.35 (0.73) |
| Week 8 | 0.85 (0.39) | 1.38 (0.17) | **2.06 (0.041)** | 0.15 (0.88) | -0.43 (0.67) | 0.35 (0.73) | 0.71 (0.48) |
| Week 12 | 0.29 (0.77) | 0.33 (0.74) | **2.33 (0.021)** | -0.20 (0.84) | 0.21 (0.84) | 0.02 (0.98) | 1.31 (0.19) |
| Follow-up | 0.37 (0.71) | -0.55 (0.58) | 1.51 (0.13) | -0.86 (0.39) | -0.50 (0.62) | -0.65 (0.52) | 0.71 (0.48) |
| **Nonparetic step end ρ_FP_** | | | | | | | |
| Baseline |  |  |  | -0.51 (0.61) | -1.65 (0.10) |  |  |
| Week 4 | 1.09 (0.28) | 1.10 (0.27) | 1.80 (0.07) | -0.53 (0.60) | -1.32 (0.19) | -0.02 (0.98) | 0.36 (0.72) |
| Week 8 | 0.79 (0.43) | 1.85 (0.07) | **3.50 (0.0006)** | 0.14 (0.89) | -0.09 (0.93) | 0.71 (0.48) | 1.71 (0.09) |
| Week 12 | 0.91 (0.36) | 0.68 (0.49) | **2.61 (0.010)** | -0.67 (0.50) | -0.70 (0.48) | -0.18 (0.86) | 1.03 (0.30) |
| Follow-up | -0.24 (0.81) | 0.28 (0.78) | 1.02 (0.31) | -0.19 (0.85) | -0.90 (0.37) | 0.36 (0.72) | 0.85 (0.40) |

Statistical results are presented for all tertiary outcome measures. Bolding indicates a significant effect (p<0.05).

**References**

1. Stimpson KH, Heitkamp LN, Embry AE, Dean JC. Post-stroke deficits in the step-by-step control of paretic step width. Gait Posture. 2019;70: 136–140. doi:10.1016/j.gaitpost.2019.03.003

2. Howard KE, Reimold NK, Knight HL, Embry AE, Knapp HA, Agne AA, et al. Relationships between mediolateral step modulation and clinical balance measures in people with chronic stroke. Gait Posture. 2024;109: 9–14. doi:10.1016/j.gaitpost.2024.01.014

3. Hoogstad LA, van Leeuwen AM, van Dieën JH, Bruijn SM. Can foot placement during gait be trained? Adaptations in stability control when ankle moments are constrained. Journal of Biomechanics. 2022;134: 110990. doi:10.1016/j.jbiomech.2022.110990
