# Supplementary material for: Effect of mediolateral leg perturbations on walking balance in people with chronic stroke: A randomized controlled trial
Source: PLoS One. 2024 Oct 8;19(10):e0311727. doi: 10.1371/journal.pone.0311727 (PMC11460716; doi:10.1371/journal.pone.0311727)
Supplement: S2 Appendix — (DOCX) [file pone.0311727.s003.docx]

**Effect of mediolateral leg perturbations on walking balance in people with chronic stroke: a randomized controlled trial**

**Appendix B**

The statistical results presented in the main text of this manuscript differ from our originally planned approach. Originally, we planned to perform a simple analysis in which a series of repeated measures 2-way ANOVA with interactions were used to identify significant effects of intervention group (Control, Assistive, and Perturbing) or time point (Week 4, Week 8, Week 12, and Follow-up) on the change scores in each of our preplanned outcome measures relative to their baseline value. In retrospect, such an approach would not have allowed us to detect differences in the intervention groups in the baseline outcome measure values, such as we observed with our primary outcome measure. Therefore, upon consultation with the study statistician, we revised to our statistical plan to a more complex linear mixed-model approach, which both allowed detection of such baseline differences, and allowed us to generate effect size estimates at each study time point.

Additionally, our original data analysis approach defined the end of steps as the time point when the velocity of the contralateral foot changed from moving posteriorly to moving anteriorly. While this approach was successful in previously tested control participants and PwCS, we noted some problems with this method with the wider range of participants included in the present study, as described in detail previously [1]. Therefore, we revised our definition of the end of a step to now correspond to the time when the velocity of the ipsilateral foot changed from moving anteriorly to moving posteriorly, as again justified elsewhere [1].

The results of our original statistical analyses are presented in Table S2. While much simpler than the results presented in the main text, the overall results are consistent. Basically, the change in our primary outcome measure (paretic ρ_SW_) relative to its baseline value differed significantly across intervention groups. None of the other outcome measures exhibited differences in the magnitude of their change across groups, although both FGA and ABC increased significantly across timepoints.

**Table S2. Original statistical approach results**

| Outcome measure | Intervention group main effect | Assessment timepoint main effect | Interaction effect |
| --- | --- | --- | --- |
| Paretic ρ_SW_ | **p = 0.047** | p = 0.77 | p = 0.53 |
| FGA | p = 0.16 | **p = 0.023** | p = 0.82 |
| ABC | p = 0.64 | **p = 0.024** | p = 0.72 |
| Overground walking speed | p = 0.92 | p = 0.076 | p = 0.73 |

Statistical results for primary continuous outcome measures, using the originally planned statistical approach. Bolding indicates a significant effect (p<0.05)

**References**

1. Howard KE, Reimold NK, Knight HL, Embry AE, Knapp HA, Agne AA, et al. Relationships between mediolateral step modulation and clinical balance measures in people with chronic stroke. Gait Posture. 2024;109: 9–14. doi:10.1016/j.gaitpost.2024.01.014
