# Supplementary material for: Effect of mediolateral leg perturbations on walking balance in people with chronic stroke: A randomized controlled trial
Source: PLoS One. 2024 Oct 8;19(10):e0311727. doi: 10.1371/journal.pone.0311727 (PMC11460716; doi:10.1371/journal.pone.0311727)
Supplement: S4 Appendix — (DOCX) [file pone.0311727.s005.docx]

**Effect of mediolateral leg perturbations on walking balance in people with chronic stroke: a randomized controlled trial**

**Appendix D**

The table below includes the mean and 95% confidence interval values plotted in the figures of the main text.

**Table S3. Group values for each timepoint and changes from baseline**

| **Outcome Metric** | Values at each timepoint: mean [95% CI] | | | Change from baseline: mean [95% CI] | | |
| --- | --- | --- | --- | --- | --- | --- |
|  | **Control** | **Assistive** | **Perturbing** | **Control** | **Assistive** | **Perturbing** |
| **Paretic ρ_SW_** | | | | | | |
| Baseline | 0.47 [0.37 0.58] | 0.41 [0.31 0.51] | **0.32 [0.22 0.41]** |  |  |  |
| Week 4 | 0.44 [0.34 0.55] | 0.42 [0.32 0.52] | 0.45 [0.36 0.54] | -0.03 [-0.13 0.06] | 0.00 [-0.09 0.10] | **0.13 [0.05 0.22]** |
| Week 8 | 0.44 [0.34 0.55] | 0.49 [0.38 0.59] | 0.43 [0.34 0.53] | -0.03 [-0.12 0.06] | 0.07 [-0.02 0.16] | **0.12 [0.04 0.20]** |
| Week 12 | 0.50 [0.40 0.61] | 0.42 [0.32 0.52] | 0.42 [0.32 0.51] | 0.03 [-0.07 0.12] | 0.01 [-0.08 0.10] | **0.10 [0.02 0.18]** |
| Follow-up | 0.45 [0.34 0.55] | 0.41 [0.31 0.51] | 0.41 [0.32 0.50] | -0.02 [-0.11 0.06] | 0.00 [-0.09 0.08] | **0.10 [0.02 0.17]** |
| **Paretic ρ_FP_** | | | | | | |
| Baseline | -0.15 [-0.27 -0.03] | -0.07 [-0.18 0.05] | -0.13 [-0.24 -0.03] |  |  |  |
| Week 4 | -0.12 [-0.24 0] | -0.09 [-0.21 0.02] | 0.02 [-0.08 0.13] | 0.03 [-0.08 0.14] | -0.03 [-0.13 0.08] | **0.15 [0.06 0.25]** |
| Week 8 | -0.14 [-0.26 -0.02] | -0.02 [-0.14 0.09] | **0.08 [-0.03 0.18]** | 0.01 [-0.09 0.12] | 0.04 [-0.06 0.15] | **0.21 [0.11 0.30]** |
| Week 12 | -0.08 [-0.20 0.04] | -0.08 [-0.19 0.04] | 0.04 [-0.07 0.14] | 0.08 [-0.03 0.18] | -0.01 [-0.12 0.09] | **0.17 [0.07 0.26]** |
| Follow-up | -0.13 [-0.25 -0.01] | -0.11 [-0.22 0.01] | 0.03 [-0.07 0.14] | 0.03 [-0.03 0.13] | -0.04 [-0.14 0.06] | **0.16 [0.07 0.26]** |
| **Paretic ρ_PD_** | | | | | | |
| Baseline | 0.81 [0.73 0.89] | 0.71 [0.63 079] | **0.65 [0.58 0.72]** |  |  |  |
| Week 4 | 0.79 [0.71 0.87] | 0.7 [0.62 0.78] | **0.66 [0.6 0.73]** | -0.02 [-0.08 0.04] | -0.01 [-0.07 0.05] | 0.01 [-0.04 0.07] |
| Week 8 | 0.78 [0.70 0.86] | 0.72 [0.64 0.79] | 0.69 [0.62 0.76] | -0.03 [-0.09 0.03] | 0.01 [-0.05 0.07] | 0.04 [-0.02 0.09] |
| Week 12 | 0.8 [0.72 0.88] | 0.72 [0.64 0.79] | **0.66 [0.59 0.73]** | -0.01 [-0.07 0.05] | 0.01 [-0.05 0.06] | 0.01 [-0.04 0.06] |
| Follow-up | 0.8 [0.72 0.88] | 0.71 [0.64 0.79] | **0.66 [0.59 0.73]** | -0.01 [-0.07 0.05] | 0.00 [-0.05 0.06] | 0.01 [-0.05 0.06] |
| **Paretic mediolateral foot placement (mm)** | | | | | | |
| Baseline | 151 [123 178] | 158 [132 185] | 178 [154 202] |  |  |  |
| Week 4 | 130 [102 157] | 160 [134 187] | 159 [135 183] | **-21 [-34 -8]** | 2 [-11 14] | **-19 [-30 -8]** |
| Week 8 | 141 [112 168] | 155 [129 182] | 157 [133 181] | -10 [-23 2] | -3 [-15 9] | **-21 [-32 -10]** |
| Week 12 | 141 [113 169] | 150 [123 177] | 155 [131 179] | -10 [-23 3] | -9 [-21 4] | **-23 [-34 -12]** |
| Follow-up | 141 [113 169] | 156 [129 183] | 158 [133 182] | -10 [-23 3] | -2 [-15 10] | **-20 [-32 -9]** |
| **FGA** | | | | | | |
| Baseline | 13.5 [10.9 16.2] | 15.5 [13 18] | 13.8 [11.5 16.1] |  |  |  |
| Week 4 | 15.2 [12.5 17.8] | 15.6 [13 18.1] | 15.2 [12.9 17.5] | **1.6 [0.3 2.9]** | 0.1 [-1.2 1.3] | **1.5 [0.3 2.6]** |
| Week 8 | 16 [13.4 18.6] | 16.3 [13.7 18.8] | 15.7 [13.4 18] | **2.5 [1.1 3.8]** | 0.9 [-0.5 2.1] | **1.9 [0.8 3.1]** |
| Week 12 | 15.6 [13 18.3] | 17 [14.5 19.5] | 16.6 [14.3 19] | **2.1 [1.1 3.8]** | **1.5 [0.2 2.8]** | **2.9 [1.7 4.0]** |
| Follow-up | 15.5 [12.8 18.1] | 16.5 [14 19] | 15.9 [13.6 18.2] | **1.9 [0.6 3.3]** | 1.0 [-0.3 2.3] | **2.2 [1.0 3.4]** |
| **ABC** | | | | | | |
| Baseline | 69 [60 79] | 66 [57 75] | 70 [62 78] |  |  |  |
| Week 4 | 73 [64 82] | 68 [59 77] | 69 [61 77] | 4 [-2 10] | 2 [-4 8] | -1 [-6 5] |
| Week 8 | 73 [64 83] | 68 [59 77] | 69 [60 77] | 4 [-2 10] | 3 [-3 8] | -1 [-7 4] |
| Week 12 | 75 [65 84] | 72 [63 81] | 73 [64 81] | 5 [-1 12] | **6 [0 12]** | 3 [-3 8] |
| Follow-up | 73 [64 82] | 74 [65 83] | 74 [66 83] | 4 [-3 10] | **8 [2 14]** | 5 [-1 10] |
| **Overground speed (m/s)** | | | | | | |
| Baseline | 0.78 [0.61 0.94] | 0.84 [0.68 1.00] | 0.73 [0.59 0.88] |  |  |  |
| Week 4 | 0.81 [0.65 0.98] | 0.85 [0.69 1.01] | 0.77 [0.62 0.91] | 0.03 [-0.01 0.08] | 0.01 [-0.03 0.06] | 0.03 [-0.01 0.07] |
| Week 8 | 0.82 [0.65 0.98] | 0.86 [0.7 1.02] | 0.77 [0.63 0.91] | 0.04 [-0.01 0.08] | 0.02 [-0.02 0.07] | 0.04 [0 0.08] |
| Week 12 | 0.83 [0.67 1.00] | 0.88 [0.72 1.04] | 0.75 [0.61 0.90] | **0.06 [0.01 0.10]** | **0.05 [0 0.09]** | 0.02 [-0.02 0.06] |
| Follow-up | 0.79 [0.63 0.96] | 0.84 [0.68 1.00] | 0.74 [0.60 0.89] | 0.01 [-0.04 0.06] | 0.01 [-0.04 0.05] | 0.01 [-0.04 0.05] |
| **Treadmill speed (m/s)** | | | | | | |
| Baseline | 0.43 [0.29 0.57] | 0.41 [0.28 0.54] | 0.37 [0.25 0.49] |  |  |  |
| Week 4 | 0.58 [0.44 0.72] | 0.48 [0.34 0.61] | 0.46 [0.34 0.58] | **0.15 [0.06 0.24]** | 0.07 [-0.01 0.15] | **0.09 [0.01 0.16]** |
| Week 8 | 0.57 [0.43 0.71] | 0.52 [0.39 0.65] | 0.52 [0.40 0.64] | **0.14 [0.06 0.22]** | **0.11 [0.04 0.19]** | **0.14 [0.07 0.21]** |
| Week 12 | 0.57 [0.44 0.71] | 0.52 [0.39 0.65] | 0.49 [0.38 0.61] | **0.14 [0.07 0.21]** | **0.11 [0.04 0.18]** | **0.12 [0.06 0.18]** |
| Follow-up | 0.54 [0.40 0.67] | 0.46 [0.33 0.59] | 0.45 [0.33 0.57] | **0.11 [0.05 0.16]** | 0.05 [0.00 0.11] | **0.08 [0.03 0.13]** |

In the first three columns of data, bolding indicates a significant difference between the metric in the indicated group and the Control group at this timepoint (p<0.05). In the final three columns of data, bolding indicates a significant change from the baseline value in that group (p<0.05).
