# Supplementary material for: Effect of mediolateral leg perturbations on walking balance in people with chronic stroke: A randomized controlled trial
Source: PLoS One. 2024 Oct 8;19(10):e0311727. doi: 10.1371/journal.pone.0311727 (PMC11460716; doi:10.1371/journal.pone.0311727)
Supplement: S1 File — (DOCX) [file pone.0311727.s006.docx]

**Official title: A novel mechanics-based intervention to improve post-stroke gait stability**

**NCT number: NCT02964039**

**PROTOCOL TITLE:**

A novel mechanics-based intervention to improve post-stroke gait stability

**PRINCIPAL INVESTIGATOR:**

Jesse Dean

# Objectives / Specific Aims

Following a stroke, quality of life is often limited by gait instability [1], due to increases in fall-risk [2-3] and fear of falling [4-5]. Recent large-scale interventions have failed to effectively address this problem [6-8], likely as a result of not being targeted to the mechanisms underlying post-stroke gait instability. This project will conduct initial testing of a novel rehabilitation method designed to improve post-stroke gait stability, based on mechanistic principles of motor learning. Specifically, this work is based on our promising preliminary work, in which weak mediolateral forces applied to the legs during walking using a novel force-field caused beneficial stabilizing after-effects in both neurologically intact controls and individuals who had experienced a stroke.

The first Aim is to identify the simplest force-field control method sufficient to modulate foot placement. We hypothesize that adjusting foot placement based solely on pelvis displacement will be sufficient to produce a more stable gait pattern. The second Aim (the clinical trial component) is to determine whether repeated gait practice with foot placement error-reduction or error-augmentation methods promotes retained use of the typical gait stabilization strategy. We hypothesize that error-augmentation methods will produce significantly larger effects than observed in either an error-reduction group or a control group. The third Aim is to quantify the effects of baseline characteristics and intervention dosage on the responses to error-reduction and error-augmentation methods. We hypothesize that lower-functioning individuals will benefit more from error-reduction, while higher-functioning individuals will benefit more from error-augmentation.

# 2.0 Background

Approximately 800,000 Americans experience a stroke every year [9]. During recovery, the restoration of gait function is a high-ranking goal for patients [10], as independent mobility is a primary factor influencing quality of life [11]. While existing gait rehabilitation methods can sometimes improve function, only about half of individuals who have experienced a stroke return to typical levels of community ambulation [12].

A major contributing factor to post-stroke reductions in mobility is gait instability, which can cause an increased risk of falls [2-3] and fear of falling [4-5]. Unfortunately, existing interventions focused on balance and strengthening [6], multifactorial fall prevention [7], and locomotor training [8] have failed to substantially impact gait instability, as evidenced by continued high fall incidence. These failures suggest the need for therapies focused on mechanisms specific to post-stroke instability [13]. For example, stroke survivors are more likely to fall due to intrinsic causes (i.e. not caused by slips, trips, pushes, etc.) than age-matched controls [2]. As recent results suggest the importance of targeting training toward the specific causes of losses of balance [14], interventions that reduce the risk of such internal perturbations may be particularly helpful among stroke survivors.

Our preliminary work suggests a path forward. We have demonstrated that neurologically intact controls use a consistent strategy of adjusting their foot placement in response to the mechanical state of their pelvis [15]. In contrast, we found that a sub-population of stroke survivors with increased fall-risk does not use this typical gait stabilization strategy, instead tending to simply place their paretic leg far laterally [16]. The most likely explanation for this altered post-stroke behavior is “learned non-use”, in which repeated avoidance of relying on the paretic limb contributes to chronic deficits [17]. Based on principles of motor learning, the typical gait stabilization strategy may thus be restored by either encouraging participants to walk with the typical gait stabilization strategy (error-reduction) [18], or by promoting error-driven adaptation through error-augmentation [19].

In preliminary work (n=15 neurologically intact controls; n=6 chronic stroke survivors), we have tested the effects of applying weak mediolateral forces to the legs during walking, pushing the legs either toward an appropriate mechanical location (error-reduction) or away from an appropriate location (error-augmentation). We found that error-reduction methods caused beneficial increases in use of the typical gait stabilization strategy while being applied. In contrast, we found that error-augmentation methods caused improvements in the use of the typical gait stabilization strategy after application, in the form of beneficial after-effects.

The purpose of this study is to reveal whether our novel force-field methods can have a beneficial effect on the gait stabilization strategy used by chronic stroke survivors. Promising results will provide the basis for larger scale clinical trials to quantify the effects on post-stroke functional mobility.

# 3.0 Intervention to be studied

Aim 2 of this study will involve the initial testing of an entirely novel intervention, to determine whether larger-scale testing is justified. Given the novelty of this intervention, no citations are available. However, the structure of this intervention is directly based on a well-known intervention (split-belt walking) that has the similar goal of improving the characteristics of walking among chronic stroke survivors [20].

No medications are involved in this intervention.

A novel mechanical device is involved in this intervention. Specifically, we have developed a force-field that is able to apply weak mediolateral forces to the legs of users while they walk. The device consists of linear actuators (i.e. motors) that are used to reposition trolleys within steel frames that are secured in front and behind a treadmill embedded in the floor of the laboratory. Nylon-coated wires in series with extension springs run from the trolley behind the treadmill to the trolley in front of the treadmill, always running parallel to the walking direction. These wires pass through leg cuffs strapped to the lateral sides of the users’ legs, with an orientation that allows completely free motion of the legs in the anteroposterior (forward/backward) and vertical directions. The only effect of the wires is to exert a weak mediolateral force on the legs when they deviate from the mediolateral position of the corresponding actuators.

While this intervention involves a novel device, we believe that it should be classified as having “Nonsignificant Risk”. Even in comparison to devices listed by the FDA as having Nonsignificant Risk (e.g. implantable vascular access devices, electrical neuromuscular stimulators, etc.), our novel device has substantially less risk. Our device is non-invasive. In fact, the actively powered components of the device (linear actuators) do not directly interface with the users at all, and are located ~10 feet away. The largest forces that our device is able to apply to users is only ~5 pounds, a force that can be easily overcome. As reference, the forces that a treadmill exerts on the legs during walking are substantially higher than this. The wires are constrained to always run laterally to the users’ legs, so there is no risk of tripping over the wires. Additionally, hard mechanical stops in the actuators prevent the wires from ever encouraging users to step off the side of the treadmill belts, or from causing cross-over steps. In summary, the novel device is entirely unable to drive users into a non-anatomical position or to directly cause a loss of balance.

While our device can thus not directly cause a fall, it is theoretically possible that stroke survivors could respond inappropriately to the forces applied, causing themselves to lose their balance. To prevent a fall in this case, participants will always wear a safety harness attached to an overhead rail. Additionally, a member of the study staff will always stand immediately next to the treadmill to provide assistance if a participant becomes uncomfortable.

Experiments using these methods of applying weak mediolateral forces to the legs during walking have previously been approved by the MUSC IRB for neurologically-intact controls (Pro00056100) and chronic stroke survivors (Pro00046775). In preliminary testing involving 15 controls and 6 stroke survivors, no participants have experienced a loss of balance or any other adverse events.

The intervention will include 54 chronic stroke survivors, divided into three groups: 1) an Error-Reduction group; 2) an Error-Augmentation group; 3) an Activity-Matched Control group. The intervention will include 24 training sessions over a 12-week period. Assessment sessions will be performed prior to beginning the intervention, every 4-weeks during the intervention, and at a 12-week follow-up. Details of the procedures are described below under Study Design / Methods.

# 4.0 Inclusion and Exclusion Criteria/ Study Population

Participants will be recruited from an MUSC database containing the contact information of stroke survivors who have agreed to be contacted for research participation (Pro00037803), and from a local VA database of stroke survivors (Pro00043107). Additionally, IRB-approved study flyers will be posted in area rehabilitation clinics and distributed through stroke support groups attended by study staff. Initial screening will be performed by study staff through a phone call to confirm basic participant characteristics (e.g. timing of stroke, ability to walk) and interest in research participation. The ability of potential participants to meet the more detailed inclusion and exclusion criteria will subsequently be determined in person.

**Inclusion Criteria** will be: 1) Age >= 21 years; 2) Experience of a stroke at least 6 months prior to participation; 3) Preferred overground gait speed of at least 0.2 m/s; 4) Ability to walk at self-selected speed for 3-minutes without a cane or walker; 5) Provision of informed consent.

**Exclusion Criteria** will be: 1) Resting heart rate above 110 beats/min; 2) Resting blood pressure higher than 200/110 mm Hg; 3) History of congestive heart failure, unstable cardiac arrhythmias, hypertrophic cardiomyopathy, severe aortic stenosis, angina or dyspnea at rest or during activities of daily living; 4) Preexisting neurological disorders or dementia; 5) History of major head trauma; 6) Legal blindness or severe visual impairment; 7) History of DVT or pulmonary embolism within 6 months; 8) Uncontrolled diabetes with recent weight loss, diabetic coma, or frequent insulin reactions; 9) Orthopedic injuries or conditions (e.g. hip replacements) with the potential to alter the ability to adjust foot placement while walking.

It should be noted that participants will be permitted to wear daily-use orthoses or braces during walking trials, such as those used to prevent equinus issues at the ankle.

This research will include women and minorities to the extent reflected by the composition of the population in Charleston and surrounding areas, and approximately matched by the demographics of the databases from which we will recruit participants. There are no inclusion or exclusion criteria that would exclude or preclude women or minorities from participating in this study.

No special classes of subjects will be included in this study. Children will not be included in the proposed study, as the mechanism of recovery from stroke in children could differ from the mechanism of recovery in adults (due to varied nervous system plasticity).

# 5.0 Number of Subjects

A total of 141 chronic stroke survivors will be accrued locally.

# 6.0 Setting

All research will be performed in the MUSC College of Health Professions Research Building at 77 President St. Charleston, South Carolina. This building is classified as shared space between MUSC and the Ralph H. Johnson VAMC. As this project will be funded by the VA, both MUSC and the Ralph H. Johnson VAMC will be considered sites for this research.

# 7.0 Recruitment Methods

As briefly mentioned above, participants will be recruited from an MUSC database containing the contact information of stroke survivors who have agreed to be contacted for research participation (Pro00037803). Participants will also be recruited from a local VA database of stroke survivors (Pro00043107). Finally, IRB-approved study flyers will also be posted in area rehabilitation clinics attended by individuals who have experienced a stroke, and will be distributed through stroke support groups attended by study staff. Potential participants will be contacted by phone to determine their interest in participation and their basic eligibility for the study.

# 8.0 Consent Process

Informed consent will be obtained from participants prior to participation. Participants will first be informed of the purpose of the experiments and possible risks. A member of the study staff with then review the Informed Consent form with the potential participant, ensuring they are given adequate time to review the document. The potential participant will be asked if they have any questions about the study, and asked if they agree to participate. The Informed Consent and HIPAA forms will be signed by the participant. Copies of the signed forms will be given to the participant. The consent process will take place in a private room in the MUSC College of Health Professions Building. There will be no set period between informing the prospective participant and obtaining the consent. In every session, participants will be reminded that they may end their participation in the study at any point.

# 9.0 Study Design / Methods

This study will consist of three Aims. Before enrollment in any of the experiments, we will first determine whether potential participants lack the typical gait stabilization strategy (the focus of this project). This will be accomplished in an initial assessment session, described in more detail below. Briefly, we will determine if each participant adjusts their mediolateral foot placement based on the mechanical state of their pelvis. We anticipate that up to a total of 141 chronic stroke survivors will be enrolled in this initial assessment session.

All assessment sessions in this study will last approximately 3 hours, and will involve the quantification of several metrics:

- Biomechanical gait characteristics. Participants will walk on an instrumented treadmill (Bertec; Columbus, OH) at their self-selected speed for 3-minutes. Active LED markers (PhaseSpace; San Leandro, CA) will be placed on participants’ legs. This equipment will allow us to quantify gait kinetics and kinematics. Of primary interest, we will use these data to quantify the correlation between pelvis mechanics at the start of a step and step width at the end of a step, our primary measure of whether an individual is using the typical gait stabilization strategy.
- Self-selected overground gait speed. Participants will perform 5 trials walking along a 10-meter straight line path at their self-selected speed, with 2 unrecorded meters for both acceleration and deceleration.
- Clinical tests. A blinded physical therapist will quantify several measures of post-stroke function commonly used clinically (e.g. Functional Gait Assessment; Activities-specific Balance Confidence scale; Fear of Falling; Fall incidence; Fugl-Meyer Lower Extremity Motor Function subscale; Fugl-Meyer Sensation subscale).

The first 12 participants from the initial assessment session who do not use the typical gait stabilization strategy will be enrolled in Aim 1. This Aim consists of a single experimental session, which will last approximately 3 hours. Participants will perform a series of treadmill trials at their self-selected speed, in which our novel force-field will produce varied assistive patterns of force. Specifically, the assisted foot placement will be based on various combinations of pelvis displacement, pelvis velocity, and average step width. As in assessment sessions, active LED markers will be placed on participants’ legs, allowing us to quantify biomechanical gait characteristics.

The next 54 participants from the initial assessment session who do not use the typical gait stabilization strategy will be enrolled in Aim 2. Of note, participants from Aim 1 will be eligible to re-enroll after a 4-week washout period. Participants will be assigned to either an Error-Reduction group (n=18), an Error-Augmentation group (n=18), or an Activity-Matched Control group (n=18). Each of these groups will complete a 12-week (24-training session) intervention. Assessment sessions will be performed every 4-weeks during the intervention, and as a 12-week follow-up.

For each training session (lasting ~1 hour), participants will perform a series of walking trials at their self-selected speed, both overground and on a treadmill. This will include ten 3-minute trials in which participants are interfaced with the force-field. The force-field will be programmed to either assist foot placement (Error-Reduction), perturb foot placement (Error-Augmentation), or simply get out of the way (Control). As in assessment sessions, active LED markers will be placed on participants’ legs, allowing us to quantify biomechanical gait characteristics.

Aim 3 involves only secondary analyses of data collected in the experiments for Aim 2.

# 10.0 Data Management

For Aim 1, we will use non-inferiority analysis methods (margin=0.10) to determine whether simpler control methods are as effective as the most complex control method at shaping foot placement. Based on our preliminary data, a power analysis found that a sample size of 12 would be sufficient for a power of over 90%.

For Aim 2, we will use a two-way repeated measures ANOVA with interactions to determine whether changes in our metric of the typical gait stabilization strategy is significantly influenced by assessment session number or group (Error-Reduction vs. Error-Augmentation vs. Control). Based on our preliminary data, a power analysis found that a sample size of 14 per group (accounting for 4 dropouts) would be sufficient for a power of over 80%.

The analyses in Aim 3 are more exploratory. We will use multivariate linear regression analyses to determine whether baseline measures of participant function can predict whether an individual at a given functional level is more likely to benefit from Error-Reduction or Error-Augmentation methods.

Steps will be taken to minimize the risk of loss of confidentiality. Clinical data from assessment sessions will be recorded on hard copies (stored in a locked file cabinet in a locked office) and entered into an electronic spreadsheet by the physical therapist immediately after each session. Gait and strength data will be analyzed using a custom MATLAB program, automatically populating a spreadsheet. The research assistant will check the data entry, and pool all data into a group-wide de-identified spreadsheet. All data from training sessions will also be analyzed and stored using an automated MATLAB program. All electronic data will be stored on a password-protected secure server that is backed-up nightly. Upon completion of Aim 2, the de-identified spreadsheets and group assignment list will be shared with the statistician for analysis. The de-identified dataset will be available for sharing with other investigators, and this trial will be registered in the NIH-supported registry.

# 11.0 Provisions to Monitor the Data to Ensure the Safety of Subjects

A Data Safety Monitoring Board (DSMB) will oversee the experiments in Aim 2 (a small-scale randomized controlled trial). This board will consist of Dr. Rick Segal (a physical therapist and principal investigator of the TIGRR program for promoting rehabilitation research), Dr. Leo Bonilha (a neurologist and stroke-recovery researcher at MUSC), and Dr. Viswanathan Ramakrishnan (a PhD level biostatistician). The DSMB will meet on a quarterly basis. The role of the DSMB will be to:

- Protect the safety of study participants
- Review the research protocol, informed consent documents, amendments, and plans for data safety and monitoring
- Evaluate the progress of the trial, including periodic assessments of data quality and timeliness, recruitment, accrual, and retention, participant risk vs. benefit, and other factors that may potentially affect study outcome
- Review study performance, make recommendations and assist in the resolution of problems reported by the Principal Investigator
- Report to the IRB on the safety and progress of the trial
- Ensure the confidentiality of the study data and the results of the monitoring
- Advise the IRB and the study investigators as to whether the protocol should continue as scheduled or undergo a modification due to a finding from the monitoring process

Any adverse events will be recorded, monitored, and promptly reported to the IRB, following policy HRPP 4.7. Our exclusion criteria will minimize the risk of enrolling participants with severe cardiovascular risk. Scheduled rest breaks will be provided between trials during experimental testing, as well as whenever requested. Minimization of risk of adverse events will be accomplished by monitoring vital signs during trials in which subjects are performing potentially demanding exercise.

# 12.0 Risks to Subjects

Potential risks for participation in this study are low.

For the assessment of biomechanical gait characteristics, participants will walk on an instrumented treadmill at their self-selected speed. To assess overground gait speed, participants will walk in a straight line across the lab. During walking, there is the risk of a loss of balance due to a trip or stumble. To mitigate this risk, in all walking trials participants will wear a safety harness attached to an overhead rail. The harness will be designed to eliminate the consequences of falling as the device “catches” the subject should they trip or stumble. The presence of this device affords comfort and diminishes fear of falling in subjects. Additionally, in all walking trials, an investigator will be near the participant to provide assistance in the event of a loss of balance. There is also the risk of minor muscle soreness due to the exercise of walking. This risk will be mitigated by walking only at each participant’s self-selected speed, and allowing rest breaks if participants ever indicate they are fatigued.

Lower extremity strength will be assessed using standard methods of dynamometric testing. During this testing, there is the risk of developing muscle soreness. To mitigate this risk, participants will perform only isometric muscle contractions (in which the muscle does not change length). These types of contractions have a lower risk of muscle damage than eccentric (lengthening) contractions. Additionally, only three short-lasting (~3 seconds) will be performed for each joint, minimizing the risk of injury due to sustained force generation.

General post-stroke function will be assessed using several commonly-used clinical tests. There is a risk of a loss of balance during the Functional Gait Assessment, in which individuals perform various functional gait tasks. We will mitigate this risk by having a physical therapist always next to participants, as is common in a clinical context. The other clinical tests either involve verbal questions or are of minimal physical risk.

In some treadmill walking trials, a novel, custom-built force-field will apply mediolateral forces to participants at about the level of the ankle. These forces will encourage participants to step to targeted locations on the treadmill. While these forces are weak, they may cause participants to perceive a loss of balance. To mitigate this risk, participants will always wear a harness attached to an overhead rail to prevent falls, and an investigator will always be directly next to the participant. There is no risk of the participant stepping off of the treadmill or of the force-field producing exceptionally larger forces, because mechanical stops built into the force-field limit the magnitude of the forces that can be produced.

All research studies have the risk of loss of participant confidentiality. Our Data Management Plan (see above) will minimize this risk.

# 13.0 Potential Benefits to Subjects or Others

This research has the potential to improve the gait stability of chronic stroke participants, which may have beneficial effects on functional mobility and quality of life. Promising effects would justify larger-scale studies able to benefit a larger number of individuals. As the risks to participants are minimal (no more than a moderate period of exercise), we believe the risks are reasonable in relation to the anticipated benefits.

# 14.0 Sharing of Results with Subjects

Results will be in the form of research data, and will not be shared with participants or others.

# 15.0 Drugs or Devices

The novel device is stored in the Locomotion and Energetics Laboratory in the MUSC College of Health Professions Research Building. This device is non-invasive, does not directly interface with participants, and can exert only low forces (~5 pounds) on participants, meeting the criteria for a Nonsignificant Risk Device. Dr. Jesse Dean, the PI on this study, developed this device.

# References

1. Jones F, Riazi A. Self-efficacy and self-management after stroke: a systematic review. Disabil Rehabil. 2011; 33:797-810.

2. Jorgensen L, Engstad, Jacobsen BK. Higher incidence of falls in long-term stroke survivors than in population controls. Depressive symptoms predict falls after stroke. Stroke. 2002; 33:542-547.

3. Langhorne P, Stott DJ, Robertson L, MacDonald J, Jones L, McAlpine C, Dick F, Taylor GS, Murray G. Medical complications after stroke. A multicenter study. Stroke. 2000; 31:1223-1229.

4. Hyndman D, Ashburn A, Stack E. Fall events among people with stroke living in the community: circumstances of falls and characteristics of fallers. Arch Phys Med Rehabil. 2002; 83:165-170.

5. Ng SSM. Contribution of subjective balance confidence on functional mobility in subjects with chronic stroke. Disabil Rehab. 2011; 33:2291-2298.

6. Dean CM, Rissel C, Sherrington C, Sharkey M, Cumming RG, Lord SR, Barker RN, Kirkham C, O’Rourke S. Exercise to enhance mobility and prevent falls after stroke: the community stroke club randomized trial. Neurorehab Neural Repair. 2012; 26:1046-1057.

7. Batchelor FA, Hill KD, Mackintosh SF, Said CM, Whitehead CH. Effects of a multifactorial falls prevention program for people with stroke returning home after rehabilitation: a randomized controlled trial. Arch Phys Med Rehabil. 2012; 93:1648-1655.

8. Tilson JK, Wu SS, Cen SY, Feng Q, Rose DR, Behrman AL, Azen SP, Duncan PW. Characterizing and identifying risk for falls in the LEAPS study. A randomized clinical trial of interventions to improve walking poststroke. Stroke. 2012; 43:446-452.

9. Mozaffarian D, Benjamin EJ, Go AS, et al. Heart disease and stroke statistics – 2015 update. A report from the American Heart Association. Circulation. 2015; 131:e29-e322.

10. Bohannon RW, Horton MG, Wikholm JB. Importance of four variables of walking to patients with stroke. Int J Rehabil Res. 1991; 14:246-250.

11. Muren MA, Hutler M, Hooper J. Functional capacity and health-related quality of life in individuals post-stroke. Top Stroke Rehabil. 2008; 15:51-58.

12. Keenan MA, Perry J, Jordan C. Factors affecting balance and ambulation following stroke. Clin Orthop Relat Res. 1984; 182:165-171.

13. Mansfield A, Aqui A, Centen A, Danells DJ, DePaul VG, Knorr S, Schinkel-Ivy A, Brooks D, Inness EL, McIlroy WE, Mochizuki G. Perturbation training to promote safe independent mobility post-stroke: study protocol for a randomized controlled trial. BMC Neurology. 2015; 15:87.

14. Grabiner MD, Crenshaw JR, Hurt CP, Rosenblatt NJ, Troy KL. Exercise-based fall prevention: can you be a bit more specific? Exer Sport Sci Rev. 2014; 42:161-168.

15. Rankin BL, Buffo SK, Dean JC. A neuromechanical strategy for mediolateral foot placement in walking humans. J Neurophys. 2014; 112:374-383.

16. Dean JC, Kautz SA. Foot placement control and gait instability among people with stroke. J Rehabil Res Dev. 2015; 52: 577-590.

17. Krakauer JW. Motor learning: its relevance to stroke recovery and neurorehabilitation. Curr Opin Neurol. 2006; 19:84-90.

18. Guadagnoli MA, Lee TD. Challenge point: a framework for conceptualizing the effects of various practice conditions in motor learning. J Mot Behav. 2004; 36:212-224.

19. Krakauer JW, Mazzoni P. Human sensorimotor learning: adaptation, skill, and beyond. Curr Opin Neurobiol. 2011; 21:636-644.

20. Reisman DS, McLean H, Keller J, Danks KA, Bastian AJ. Repeated split-belt treadmill training improves poststroke step length asymmetry. Neurorehab Neural Rep. 2013; 27:460-468.
