# Supplementary material for: Effect of mediolateral leg perturbations on walking balance in people with chronic stroke: A randomized controlled trial
Source: PLoS One. 2024 Oct 8;19(10):e0311727. doi: 10.1371/journal.pone.0311727 (PMC11460716; doi:10.1371/journal.pone.0311727)
Supplement: Attachment — Submitted filename: Response to Reviewers.docx [file pone.0311727.s007.docx]

**Response to Reviewer Comments**

We thank the reviewers for their thoughtful comments, which we believe have helped us to improve this manuscript. Our responses to individual comments are below in red indented text, with quotes from the manuscript italicized. All line numbers refer to the resubmitted manuscript without tracked changes.

**Reviewer #1:** The purpose of this study was to compare the effects of walking training with no forces vs. assistive forces vs. perturbing forces on step width modulation in individuals with chronic stroke. They found that the perturbing forces tended to yield changes in biomechanical outcomes, whereas this was less common in the other two groups. Overall, the manuscript is well-written with good rationale for the approach. The methods employed also appear to be appropriate for the study. I have some concerns, with the most pressing being some limitations of the statistical approach. My major and minor concerns are listed.

Major comments
• The primary weakness I see is that the statistical methods are somewhat lacking. I appreciate the transparency about the original analysis plan and the reasons it was changed. However, the approach used (unless I misunderstand) does not provide insight into any group X time interactions. On Page 14, Line 283-286, you state that the analysis focused on within-group changes across time and on between group comparisons at each time point. This will not provide insight into whether one intervention led to greater changes in your outcomes than another. However, the results imply that you are providing insight into interactions (e.g., Page 17, line 344-345, “exhibited larger increases.” So, first, please clarify whether your statistical approach looked at group X time interactions or compared change values at each time point. If the statistical design was not designed in either of these ways, the results and discussion should not be focused on interactions. For example, if the statistical approach is as described on Page 14, the presentation of change values in Table 2 is misleading. It makes it seem that the statistical tests show a greater change in the Perturbing group, when in fact, this was not tested.

In retrospect, we agree with the reviewer that our description of the study’s statistical methods (and their relationship with the study’s hypotheses and exploratory goals) was not sufficiently clear. To address this weakness, we revised the last paragraph of the Introduction to more explicitly state the hypotheses of this early-stage study, as well as which comparisons were considered exploratory:

[Lines 71-81] *“Our primary hypothesis was that repeated exposure to appropriately targeted perturbations would cause sustained increases in paretic step width modulation (**ρ_SW_) among PwCS. In contrast, we did not expect that repeated exposure to either assistance or minimal external forces (a control group) would evoke such changes in paretic ρ_SW_. Exploratory analyses compared changes in paretic ρ_SW_ between the control group and both the perturbing and assistive groups. Secondarily, we hypothesized that repeated perturbations would cause sustained increases in paretic foot placement modulation relative to the pelvis, as well as improvements in clinical assessments related to walking balance and confidence (FGA score, ABC score, and fear of falling). As with our primary metric, we performed exploratory comparisons of these outcomes (as well as real-world falls during the 12-week intervention and a 12-week follow-up period) between the control group and both the perturbing and assistive groups.”*

Our statistical comparisons were designed to both address our hypotheses (within-group changes over time) and provide insight into our exploratory comparisons (between-group differences in the magnitude of change over time). We have revised our Statistical Methods section to now state this more clearly, as well as to provide justification for focusing our hypotheses on within-group comparisons:

[Lines 304-311] *“Our statistical approach was designed with the primary goal of determining whether the outcome measures of interest changed significantly over time within each intervention group, thus addressing our primary and secondary hypotheses. This focus was based on the early stage of the research, as no prior work has investigated whether repeated exposure to this type of mechanical environment has any sustained effects on walking behavior or balance. Our approach also included an exploratory component, in which changes in outcome measures over time were compared between the Control group and the Perturbing and Assistive groups. Promising results from these analyses would provide justification for a larger-scale future investigation.”*

[Lines 328-334] *“For interpretation of the model results, we focused on three sets of comparisons. First, we assessed whether each outcome measure changed across Assessment sessions (time points) for each intervention group (Control, Assistive, and Perturbing), addressing our primary and secondary hypotheses. We then assessed whether each outcome measure differed from the Control group at each time point (for the Assistive and Perturbing groups). Finally, we assessed whether the change in each outcome measure from its baseline value differed from the Control group at each subsequent time point (for the Assistive and Perturbing groups), addressing our exploratory questions.”*

We also now explicitly state in the Results section which of the presented findings are derived from our exploratory analyses.

• My guess is that you did not have enough power to test for interaction effects for several reasons. 1) your sample size calculation was based on 2, not 3 groups. 2) the large number of timepoints included in the model reduced the number of degrees of freedom. This may be why you don’t state a hypothesis about one intervention compared to another despite the introduction clearly implying that the error augmentation group will have greater improvements/changes. I think the best solution to this problem would be to revise the statistical approach to have two timepoints (Pre/Post) or maybe 3 (Pre/Post/FU). If this approach were used, you could explain the lack of power to test for interactions across 5 time points. Because the pandemic interrupted the study, I think you also have a justifiable rationale for not collecting data from more participants.

We hope that more clearly distinguishing between our hypotheses and exploratory analyses for this early-stage study (see answer above) helps to address this concern. For the reasons cited by the reviewer (e.g., sample size), our primary focus is on within-group changes in our outcome measures, as we are more likely to have sufficient statistical power for such comparisons than for interaction-type effects between intervention groups.

Throughout the manuscript, we now emphasize the specific goal of this study (focusing on within-group changes):

[Lines 57-58] *“The purpose of this study was to investigate whether the modulation of mediolateral paretic step location can be strengthened through repeated exposure to a novel mechanical environment…”*

[Lines 71-74] *“Our primary hypothesis was that repeated exposure to appropriately targeted perturbations would cause sustained increases in paretic step width modulation (ρ_SW_) among PwCS. In contrast, we did not expect that repeated exposure to either assistance or minimal external forces (a control group) would evoke such changes in paretic ρ_SW_.”*

[Lines 304-306] *“Our statistical approach was designed with the primary goal of determining whether the outcome measures of interest changed significantly over time within each intervention group, thus addressing our primary and secondary hypotheses.”*

[Lines 481-483] *“The study results only partially support our hypotheses, as paretic step width modulation, mediolateral paretic foot placement modulation, and FGA score all increased relative to baseline in the Perturbing intervention group.”*

[Lines 496-499] *“However, our exploratory statistical comparisons did not consistently support the stronger claim that the Perturbing group exhibited larger effects than the Control group, likely due in part to the higher-than-expected attrition rate in the Control group (28%).”*

With respect to the inclusion of multiple time points, our preference is to include the results from all assessments in our analyses, as the time course of potential changes in gait evoked by the delivered perturbations was entirely unknown prior to this study. By including all time points, readers can gain initial insight into this time course, which could be relevant for designing future intervention studies of this type. For example, the results suggest that biomechanical effects are present after only 4-weeks of training, and do not appear to increase further with additional training.

• The use of a per-protocol analysis instead of intention-to-treat is potentially an issue because you lost more people from the assistive group than the main comparator group, perturbing. If my other comments are correct about the statistical analysis, I recommend revising the statistical approach to an intention-to-treat model, probably with a missing-at-random approach.

We appreciate the reviewer’s point, but believe that a per-protocol (PP) analysis is more appropriate than an intention-to-treat (ITT) analysis at this stage of this line of research. In our interpretation, a PP analysis addresses the question “What is the effect of receiving an intervention in a group of patients?”, while an ITT analysis addresses the question “What is the effect of assigning an intervention to a group of patients?”. While the latter question would be more relevant for determining the clinical effectiveness of an intervention, we believe that in the current early stage it is more important to determine whether the perturbation intervention under investigation has the potential to have beneficial effects if delivered as intended.

In our opinion, the reasons for participant dropout support our use of a PP analysis. Eight of the ten participants who did not complete the protocol did so due to either the COVID-19 shutdown or because of medical reasons unrelated to the intervention. We had minimal dropout due to unknown reasons (loss of contact) which could have feasibly been related to the intervention itself.

We have added a brief justification for our use of a per-protocol analysis to the Statistical Methods section:

[Lines 312-316] *“Given the early stage of this research, we performed per-protocol analyses that included participants who completed all Assessment sessions. This approach allowed us to focus on the effects in participants who received the prescribed interventions, rather than those whose participation ended due to reasons other than the interventions themselves. However, this approach prevents us from making direct claims regarding clinical effectiveness.”*

Minor comments
Overall
• One issue throughout the text is the nomenclature used to designate the different experimental groups. Specifically, the word perturbing is used for one group, but both the assistive and perturbing group experience perturbations. I believe interpretation of the text would be improved if you used to nomenclature on ClinicalTrials.gov: “error reduction,” “error augmentation,” and “control.”

As noted by the reviewer, the original terminology we used in this project was “error reduction” and “error augmentation”. However, this terminology was criticized by an earlier set of reviewers, who argued that our methods do not augment errors in such a way that is “deemed deterministic by the subjects”, and thus the phrase error augmentation should not be used. Since that time, we have used the terms Perturbing and Assistive in several publications (Heitkamp et al. 2019; Reimold et al. 2020 and 2021; van Leeuwen et al. 2024). Therefore, our preference is to keep this terminology. We have added a reference to these other terms for the action of the force-field to the Methods section:

[Lines 166-168] *“This study involved three force-field control modes described previously [10]: Transparent (previously also termed Control), Assistive (previously also termed Error Reduction), and Perturbing (previously also termed Error Augmentation).”*

In addition, we acknowledge that the Assistive group also experienced forces that they may perceive as perturbations. We have added a statement to the Methods explicitly stating why this force-field control paradigm was termed Assistive:

[Lines 172-180] *“In the Assistive mode, at the start of each step, our data collection system predicted a mechanically appropriate step width based on the mediolateral pelvis displacement from the stance foot, following speed-dependent relationships observed in neurologically-intact controls without balance deficits [6,12]. These predictions accounted for each individual participant’s baseline average step width, such that participants would not be encouraged to walk with either overly narrow or overly wide steps outside of their normal step width range [12]. The force-field then pushed the swing leg mediolaterally toward the position that would achieve this step width. Therefore, users experienced forces that assisted their performance of the targeted gait behavior, albeit potentially in the form of foot placement locations that differed from their baseline gait pattern.”*

Abstract
• From the abstract, it wasn’t clear what the overall training was, just what the different interventions were. Please add detail so readers can gather the overall intervention just from the abstract.

We have added more information about the training, while ensuring we stayed within the Abstract word limit:

[Lines 23-25] *“All intervention groups included 24 training sessions over 12-weeks with up to 30-minutes of treadmill walking while interfaced with a novel force-field and a 12-week follow-up period, with five interspersed assessment sessions.”*

Introduction
• Overall, the introduction provides a good rationale for the study.

Thank you for the supportive comment.

• No clear hypothesis is presented in the final paragraph of the Introduction. Please revise.

We believe that this comment is addressed by our response to the first reviewer comment about the statistical approach, and the associated changes to the final paragraph of the Introduction. Our revisions now more differentiate between our hypotheses (which are limited for this small-scale, early stage work) and our exploratory analyses.

In our view, keeping our hypotheses limited is reasonable, as no prior work has tested whether any intervention can have a positive sustained effect on step width modulation in this population.

Methods
• Make sure details match those on ClinicalTrials.gov.

We have reviewed the details on ClinicalTrials.gov, and ensured that deviations from the original plan are noted in the manuscript (e.g., we explicitly state which outcome measures were not preplanned, describe the change in the planned statistical analyses, and note a change in the exclusion criteria).

• Page 7, Line 134: How was it determined how many 3-minute trials they performed?

Participants performed a maximum of 10 3-minute trials in which they interfaced with the force-field, but performed fewer if any of our termination criteria were met. This is now clarified in the Methods:

[Lines 146-149] *“Treadmill trials were terminated if participants reported being too fatigued to continue, were unable to keep up with the treadmill speed, or if blood pressure during rest periods exceeded 200/110 mm Hg. Therefore, the maximum total training time of 30 minutes was not achieved in all sessions.”*

• Page 8, Line 163-164: The application of external force in the study depended on the prediction of a mechanically appropriate step width (see reference 6, 12). Presumably, there is potential for error with this prediction. Can you provide any insight?

This is an interesting question, with an answer that depends on the intended meaning of the term “error”. It is absolutely true that even neurologically-intact control participants do not walk with a perfect linear correlation between pelvis displacements and step widths. So the difference between the prediction made by the linear fit and the actual step width could be considered an “error”, although it’s unclear whether this error should be considered to be on the part of the prediction or on the part of the human who did not achieve the predicted step width.

In people with stroke, it is likely that many of the predicted mechanically appropriate step widths will be quite different from those that these individuals would achieve on their own. But we would attribute this difference (or “error”) not to the predictions being wrong, but to the participant not using a step width modulation strategy that is generally accepted to be beneficial for ensuring walking balance.

We believe that the manuscript addition cited above regarding the nature of the provided assistance will help to ensure that readers are aware of this factor:

[Lines 178-180] *“Therefore, users experienced forces that assisted their performance of the targeted gait behavior, albeit potentially in the form of foot placement locations that differed from their baseline gait pattern. “*

• Please report how many in each group had external force applied to narrow vs. widen step width. Although the interventions were designed to either assist in achieving a mechanically appropriate step width or exacerbate errors in step width, this was again using model predictions, which may err. At the same time, we know that the external forces either narrowed or widened the step. Hence, your results could have occurred because the experimental groups differed in whether the step was narrowed or widened and the variability of such.

This is an important point, as all users in both the Assistive and Perturbing groups experienced forces that encouraged narrower step widths for some steps, but encouraged wider step widths for other steps. Following the methods described in detail in a preceding paper [Reimold et al. 2021], the predictions of the mechanically appropriate step width for each participant included their baseline average step width. Therefore, the force-field did not consistently encourage either narrower or wider steps. Instead, the force-field encouraged either relatively narrow or relatively wide steps (within that participant’s normal range) based on the pelvis displacement at the start of that step. We have clarified this in the Methods:

[Lines 172-177] *“…at the start of each step, our data collection system predicted a mechanically appropriate step width based on the mediolateral pelvis displacement from the stance foot, following speed-dependent relationships observed in neurologically-intact controls without balance deficits [6,12]. These predictions accounted for each individual participant’s baseline average step width, such that participants would not be encouraged to walk with either overly narrow or overly wide steps outside of their normal step width range [12].”*

• Can you provide rationale for why the last minute of each trial was in transparent mode? Normally a “catch” trial would capture the first few steps when the perturbation was removed. Instead, 1/3rd of the intervention time was not actually the intervention.

First, as clarification, the force-field was in Transparent mode for the last minute of alternating trials (not every trial) in the Assistive and Perturbing groups. So ~1/6^th^ of the intervention time did not involve receiving assistive or perturbing leg forces, rather than 1/3^rd^:

[Lines 187-188] *“In alternating trials (starting with the second force-field trial), the force-field changed to Transparent mode for the last minute of the 3-minute walking period (see Fig 1B).”*

We have now added more detailed justification for why these catch periods were included:

[Lines 189-193] *“These “catch periods” were included for two reasons. First, their inclusion will allow future exploratory analyses to investigate the time course of potential changes in step width modulation during training sessions. Second, retention of a learned movement pattern may be enhanced when exposure to a novel mechanical environment includes periods in which this novel environment is removed, as recently observed for PwCS experiencing pelvis perturbations while walking [19].”*

• So, if they missed a session, it wasn’t just pushed to the end to ensure they achieved 24 training sessions? Can you please explain why the 24 training sessions had to be performed within a certain timeframe without flexibility?

While the ideal situation would be one in which all participants attended all 24 training sessions in the planned 12-week period, this was not always possible for various reasons (participant sickness, travel, hurricanes, etc.). As pointed out by the reviewer, the two most reasonable strategies would be to: 1) Keep the 12-week duration consistent, and complete as many training sessions as possible (max of 2 per week) in this time period; or 2) Complete 24 training sessions, no matter how long it takes.

Both options have scientific pros and cons, as both the number of sessions attended and the time frame over which the intervention is received could reasonably influence the effects of the intervention. Our choice was largely based on a practical consideration: The local IRB strongly prefers interventions with a pre-established, set duration that cannot theoretically last indefinitely.

Tables & Figures
• Table 2: please redo to show the actual values (including baseline). This will aid the reader in looking for baseline differences and not misrepresent the results.

The data requested here is currently presented in the figures, while the tables include only the results of the statistical analyses. Our understanding is that it is frowned upon to include the same data in both Figures and Tables in a manuscript (a practice that is explicitly banned in some journals). We hope it’s an acceptable compromise to add the requested table as Supplementary information (Appendix D).

• Figure 1: The presentation of transparent, assistive, and perturbing keys within the figure makes it look like the groups do 3 minutes of each, then 3 more minutes. I recommend moving this to the legend or a small legend.

The reviewer’s interpretation of this figure’s appearance is correct. For example, participants in the Assistive group perform a 3-minute trial in which the force-field is in Assistive mode. They then perform a 3-minute trial in which the force-field is in Assistive mode for the first 2 minutes and Transparent mode for the final minute. This is now clarified in the Figure legend:

[Lines 131-132] *“For both the Assistive and Perturbing groups, the force-field was in Transparent mode for the final minute of even numbered trials.”*

• Figure 2: how is medical exclusions different from exclusion criteria?

We have now clarified in the Figure legend how these medical exclusions were defined, and what they entailed:

[Lines 362-369] *“Prior to randomization, five individuals were excluded for medical reasons that were not part of our pre-planned exclusion criteria, but would have affected their ability to participate in the intervention: upcoming arm surgery, upcoming toe amputation, upcoming Achilles tendon surgery, anticipated Botox treatments, and undisclosed upcoming medical treatment. Three participants were medically withdrawn from the Control group during intervention for the following reasons: wrist injury from a car accident, experience of a stroke, hospitalization for pneumonia. One participant was medically withdrawn from the Assistive group during intervention due to being diagnosed with Parkinson’s disease. One participant was medically withdrawn from the Control group during the follow-up period due to experiencing a stroke.”*

• Figure 3 is good. It does look there may be some baseline differences between groups, hence, as discussed the statistical approach needs to be adjusted, or the manuscript needs to refrain from making between group comparisons (of changes).

The reviewer is correct that despite participant randomization, the baseline value of our primary outcome measure (paretic ρ_SW_) was lower in the Perturbing group than in the Control group. This issue is explicitly noted in the Discussion as a reason for caution in attributing the observed change in this metric to the Perturbing intervention itself:

[Lines 503-506] *“Finally, the average baseline value of paretic ρ_SW_ was notably lower in the Perturbing group than the Control group, despite participant randomization. This baseline difference may have made it more likely to observe an increase over time in the Perturbing group due simply to regression to the mean.”*

To more directly address this concern, we have added an unplanned, post-hoc statistical analysis to investigate whether the lower baseline paretic ρ_SW_ value in the Perturbing group was the source of the observed increase with subsequent intervention. We acknowledge that there are multiple statistical approaches that can be used to account for differing baseline values, each with their own assumptions about the underlying structure of the data. We here chose the simplest approach. Based on visual inspection of Figure 3A, it is clear that the lower baseline (Pre) value in the Perturbing group is caused by four participants with substantially lower metric values than any participants in the other groups. Therefore, our post-hoc statistical analysis removed these four participants to determine whether an increase in paretic ρ_SW_ was still observed in the Perturbing group once all groups had similar baseline values. Of note, after this removal, the three groups had similar number of participants included in the analyses (13 or 14). This analysis and the results are described in the Discussion:

[Lines 505-514] *“This baseline difference may have made it more likely to observe an increase over time in the Perturbing group due simply to regression to the mean. To explore this possibility, we performed a post-hoc analysis in which we removed the four participants in the Perturbing group with low outlier values for the baseline paretic ρ_SW_ metric (see Fig. 3A). Following the general statistical approach described in the Methods, we used linear mixed effect models to test whether each intervention group exhibited a significant increase in paretic ρ_SW_, focused on a comparison between baseline and all later timepoints, given the lack of apparent changes from Week 4 to Follow-Up. Here, the baseline values of paretic ρ_SW_ were similar across groups, and only the Perturbing group exhibited a significant increase in this metric at later timepoints (Table 5). Therefore, the change in paretic ρ_SW_ for the Perturbing group does not appear to be solely due to having a lower baseline value.”*

***Table 5. Primary outcome measure with baseline outliers removed.***

|  | ***Control*** | ***Assistive*** | ***Perturbing*** |
| --- | --- | --- | --- |
| *Baseline: mean [95% CI]* | *0.47 [0.38 0.57]* | *0.41 [0.34 0.49]* | *0.41 [0.34 0.49]* |
| *Change: mean [95% CI]* | *-0.015 [-0.080 0.050]* | *0.021 [-0.053 0.094]* | ***0.091 [0.026 0.156]*** |
| *Change: t (p)* | *-0.47 (0.64)* | *0.58 (0.57)* | ***2.81 (0.007)*** |

*Statistical results are presented for paretic ρ_SW_ measures after four outliers from the Perturbing group are removed. Bolding indicates a significant effect (p<0.05).*

**Reviewer #2:** This is a well-written description of a small, three-arm trial. I have a few minor comments.
1. The randomization block size was not specified. Were blocks randomized? What strategies were implemented to mitigate selection or ascertainment bias?

We have clarified the randomization method:

[Lines 290-293] *“Participants were randomly assigned to one of the three intervention groups using a sequence determined before study onset. The permuted block randomization sequence had a block size of 18, was created by a statistician otherwise not involved with the study procedures, and was stored in a secure online database.”*

We acknowledge that this study (as with nearly all research studies) includes the risk of selection bias, as individuals who volunteer for research studies may differ from the general population. We sought to reduce this potential bias by our choice of inclusion and exclusion criteria, as now directly stated in the Methods:

[Lines 96-100] *“The study inclusion and exclusion criteria were chosen to include a broad range of post-stroke individuals likely to receive physical therapy focused on walking balance (e.g., having some walking ability), while simultaneously reducing the risk of negative health events that could be caused by participation in a study that involves periods of exercise.”*

Our steps to mitigate ascertainment bias are included in the Blinding section:

[Lines 297-299] *“Clinical assessors were also blinded to the intervention group and were not in the laboratory during training sessions to ensure they did not become aware of the intervention group.”*

[Lines 300-302] *“All biomechanical metrics were calculated using standard automated code that did not include group assignment as an input.”*

2. Were the assumptions underlying the sample size (accrued from a pilot study of n=56) realized? This should be clearly stated in the Discussion, as either a strength or a weakness.

To clarify, the sample size chosen for this early stage study was based on preliminary results from 6 participants:

[Line 281] *“This sample size was based on preliminary results (n=6)…”*

We have revised the Discussion to more explicitly state that the *sustained* effects of the perturbations observed in the present work were smaller than the *immediate* effects of similar perturbations observed earlier:

[Lines 499-503] *“Additionally, the power analysis used to choose the sample size for this study was based on the effect size of the increases in paretic ρ_SW_ that immediately followed exposure to force-field perturbations (Cohen’s d of 1.3 in initial preliminary data, and 1.0 based on subsequent published work [12]). In contrast, repeated exposure to perturbations here produced a notably smaller sustained effect size of 0.6 (across time points), reducing the likelihood of detecting significant effects.”*

**Reviewer #3:** This parallel, double-blinded RCT looked at the effects of perturbation training, a control condition, and assistive training on step modulation in PwCS. The perturbation group demonstrated improvements in paretic step width modulation which were sustained through a 12 week follow-up, whereas no change was noted for the control or assistive group. Despite promising results, the authors do a great job of explaining that there was not always a difference between the perturbation and control group, therefore the changes noted may have been due to a reduction in step width modulation at baseline for the perturbation group. This study demonstrates sound research design with good reasoning and interpretation of results. I have only minor concerns that can be easily clarified by the authors.

We thank the reviewer for their kind comments, and are glad that the reviewer agrees that our caution in interpreting these early promising results is warranted.

Introduction:
- Please clarify the authors’ hypotheses regarding the ‘assisted’ and ‘minimal external forces’ conditions? It’s mentioned that authors hypothesized the perturbations would cause a change in paretic step width modulation, but nothing about how this intervention would compare to the other two.

We have revised the final paragraph of the Introduction based on this comment and the comments of Reviewer 1. We hope that our revisions more clearly differentiate between the primary hypotheses we chose to test, and the analyses that were intended to be exploratory. Just to provide the reviewer with a little more context – these choices were made based on discussions with experienced stroke rehabilitation researchers before this clinical trial began. With the sample size of this early-stage work, these researchers cited a risk that sufficient power may not be present to detect significant interaction effects between the three arms. This type of result due to potential underpowering was cited as a possible disservice to the field, as otherwise promising approaches may be discarded because of a lack of statistical significance.

Based on this feedback, we made the decision to focus our hypotheses on within-group comparisons – did the outcome measures of interest change significantly over time with respect to baseline within each group? Given the anticipated interest in comparisons between groups, we then performed planned exploratory analyses to compare each of the experimental groups to the control group.

We have now clarified this approach in the Introduction:

[Lines 71-81] *“Our primary hypothesis was that repeated exposure to appropriately targeted perturbations would cause sustained increases in paretic step width modulation (ρ_SW_) among PwCS. In contrast, we did not expect that repeated exposure to either assistance or minimal external forces (a control group) would evoke such changes in paretic ρ_SW_. Exploratory analyses compared changes in paretic ρ_SW_ between the control group and both the perturbing and assistive groups. Secondarily, we hypothesized that repeated perturbations would cause sustained increases in paretic foot placement modulation relative to the pelvis, as well as improvements in clinical assessments related to walking balance and confidence (FGA score, ABC score, and fear of falling). As with our primary metric, we performed exploratory comparisons of these outcomes (as well as real-world falls during the 12-week intervention and a 12-week follow-up period) between the control group and both the perturbing and assistive groups.”*

As mentioned by this reviewer, because of our chosen hypotheses, we have attempted to be conservative throughout the rest of the manuscript in terms of how comparisons between groups should be interpreted. Our view is that our current results indicate that our perturbation results are promising in terms of evoking changes in targeted biomechanical characteristics of the post-stroke gait pattern, but should not be seen as definitive.

Materials and Methods:
- For clarity, it would help to have something signaling that transparent mode was the concluding minute of every other trial either in Figure 1 or in the caption

Thank you for this comment. This point of confusion was also mentioned by Reviewer 1. We now more clearly describe this element of the training sessions in the figure caption:

[Lines 131-132] *“For both the Assistive and Perturbing groups, the force-field was in Transparent mode for the final minute of even numbered trials. “*

- More explanation of Paretic pPD is required for reader clarity (lines 217-222)

We agree that more explanation of this metric is warranted, and have added some more detail:

[Lines 235-244] *“We additionally calculated the partial correlation between the final mediolateral pelvis displacement at the end of a step and pelvis displacement at the start of the step, again accounting for pelvis velocity. This metric quantifies the extent to which frontal plane stance leg posture upon step onset is related to stance leg posture at the end of the same step, and can likely be partially attributed to the stance leg acting as an inverted pendulum [8]. For the purposes of the present study, this metric essentially provides insight into whether factors other than mediolateral foot placement affect our primary measure of step width modulation. This metric of stance leg position modulation was previously found to not be affected by exposure to our novel force-field [11]. This metric was added to our outcome measures to provide further insight into possible contributors to altered step width modulation.”*

Results:
- Were statistical analyses completed to determine potential significant differences in participant characteristics?

No, such analyses were not completed. Based on our understanding of the 2010 Consort guidelines, such statistical comparisons are not recommended. From Moher et al. 2010: “Such significance tests assess the probability that observed baseline differences could have occurred by change; however, we already know that any differences are caused by chance. Tests of baseline differences are not necessarily wrong, just illogical [211]. Such hypothesis testing is superfluous and can mislead investigators and their readers.”

Discussion:
- Lines 463-469 speculate that perhaps PwCS learn early in the early post-stroke period to walk with a wider base gait, and that practicing with perturbations allows them to realize that a more narrow gait is possible. However, there is no report of baseline step width listed. Was it the case that these participants demonstrated a larger than average step width during the pre-intervention assessment? And did it reduce post-intervention or at follow-up? If this wasn’t calculated, please clarify.

First, we’ve clarified this speculation in the Discussion, which focuses on consistently lateral foot placement for paretic steps, rather than consistently wide step widths. Perhaps counter-intuitively, these two measures are not always strongly linked in PwCS (e.g., see Balasubramanian et al. 2010; Clin Biomech). Many PwCS adopt gait patterns in which the paretic swing foot is placed quite laterally, while the pelvis/CoM remains fairly close to the stance foot mediolaterally, thus having only a small effect on step width.

[Lines 537-542] *“Speculatively, perhaps the baseline gait pattern with limited paretic step modulation was a habitual behavior learned in the early post-stroke period. A reduced ability to accurately control motion of the paretic swing leg may encourage a strategy in which the paretic foot is consistently placed quite lateral from the pelvis in order to prevent lateral losses of balance during the subsequent single stance phase [21], despite the accompanying increased mechanical demands [29,30].”*

With that said, we agree with the reviewer that it would be of value to present data to either support or refute this speculation. We have thus added another secondary biomechanical outcome measure of paretic mediolateral foot placement:

[Lines 246-252] *“As described above, paretic mediolateral foot placement was defined as the mediolateral distance from the sacrum to the paretic swing heel when the paretic foot lands at the end of a step. In many PwCS, paretic steps are placed far laterally relative to the pelvis [7,20], which may be a strategy intended to reduce the risk of lateral losses of balance that could occur with overly medial foot placement [21]. This metric was added to our outcome measures after study initiation to determine whether potential increases in foot placement modulation are accompanied by a willingness to walk with more medial foot placement.”*

The changes in paretic mediolateral foot placement are detailed in the Results section and have been added to Figure 3 and Table 2. Briefly, we found that as with our measures of paretic step modulation (ρ_SW_ and ρ_FP_), paretic foot placement only exhibited a consistent change relative to baseline at all subsequent time points for the Perturbing group. Average foot placement became more medial by Week 4 and remained more medial in later assessments. However, the Control group also exhibited a smaller decrease in foot placement, and no significant differences between the Control and Perturbing groups were present for the change in foot placement.

We now refer to this result in the Discussion:

[Lines 542-545] *“Repeated periods in which the delivered perturbations elicited greater active step modulation may have prompted participants to recognize that this was a gait pattern that could be less demanding and more stable, promoting its continued use. The accompanying shift in average paretic foot placement to a less lateral location is consistent with this possibility.”*

- What are your thoughts as to why there was a change in pSW for the perturbation group and not the assistance or control group for this specific intervention, when other studies you’ve sited (lined 469-475) have shown that both exaggerating and reducing errors or resistance can improve asymmetry in PwCS?

This is a great question, and we have added some speculation to the Discussion as to a possible reason for this discrepancy:

[Lines 552-559] *“One possible explanation for the unique role of perturbations in the present study is the important role of perceived imbalance in eliciting locomotor adaptation [33]. Perhaps only the Perturbing group perceived the force-field as challenging their balance and changed their gait pattern accordingly, while the Assistive and Control groups had no such motivation to adapt. In contrast, post-stroke walking balance appears to be challenged not just by augmenting step length asymmetry, but also by interventions that reduce step length asymmetry [34]. The threat of a loss of balance may be an important component of encouraging an individual to change their habitual walking pattern.”*
